# COVID-19 Risk Mapping with Considering Socio-Economic Criteria Using Machine Learning Algorithms

**DOI:** 10.3390/ijerph18189657

**Published:** 2021-09-14

**Authors:** Seyed Vahid Razavi-Termeh, Abolghasem Sadeghi-Niaraki, Farbod Farhangi, Soo-Mi Choi

**Affiliations:** 1 Geoinformation Technology Center of Excellence, Faculty of Geodesy and Geomatics Engineering, K.N. Toosi University of Technology, Tehran 19697, Iran; vrazavi70@gmail.com (S.V.R.-T.); farbod.farhangi1995@gmail.com (F.F.); 2 Department of Computer Science and Engineering, and Convergence Engineering for Intelligent Drone, Sejong University, Seoul 143-747, Korea; smchoi@sejong.ac.kr

**Keywords:** COVID-19 crisis, data-driven algorithms, geographic information system (GIS), spatial modeling, health geography

## Abstract

The reduction of population concentration in some urban land uses is one way to prevent and reduce the spread of COVID-19 disease. Therefore, the objective of this study is to prepare the risk mapping of COVID-19 in Tehran, Iran, using machine learning algorithms according to socio-economic criteria of land use. Initially, a spatial database was created using 2282 locations of patients with COVID-19 from 2 February 2020 to 21 March 2020 and eight socio-economic land uses affecting the disease—public transport stations, supermarkets, banks, automated teller machines (ATMs), bakeries, pharmacies, fuel stations, and hospitals. The modeling was performed using three machine learning algorithms that included random forest (RF), adaptive neuro-fuzzy inference system (ANFIS), and logistic regression (LR). Feature selection was performed using the OneR method, and the correlation between land uses was obtained using the Pearson coefficient. We deployed 70% and 30% of COVID-19 patient locations for modeling and validation, respectively. The results of the receiver operating characteristic (ROC) curve and the area under the curve (AUC) showed that the RF algorithm, which had a value of 0.803, had the highest modeling accuracy, which was followed by the ANFIS algorithm with a value of 0.758 and the LR algorithm with a value of 0.747. The results showed that the central and the eastern regions of Tehran are more at risk. Public transportation stations and pharmacies were the most correlated with the location of COVID-19 patients in Tehran, according to the results of the OneR technique, RF, and LR algorithms. The results of the Pearson correlation showed that pharmacies and banks are the most incompatible in distribution, and the density of these land uses in Tehran has caused the prevalence of COVID-19.

## 1. Introduction

In December 2019, an acute respiratory syndrome was reported in Wuhan, Hubei Province, China, due to the release of a new unknown virus called COVID-19. Very soon after this, new cases were identified all over China and around the world. COVID-19 spreads relatively rapidly compared to SARS-CoV in 2002–2003 and MERS-CoV in 2012–2014. While the number of patients with MERS reached 1000 in about 30 months and the number of patients with SARS reached 1000 in approximately months, the number of COVID-19 patients reached 1000 in only 48 days [1]. COVID-19 spreads so fast that it was alarmingly declared a global epidemic by the World Health Organization (WHO) on 11 March 2020 [2]. As of 4 May 2020, more than 3,435,894 people have been infected worldwide, and it can be concluded that COVID-19 has spread all around the world [3].

COVID-19 is a new virus, and little is known about it [4]; additionally, the transmission of the virus is a complex process. For this process, algorithms can be developed to predict the outbreak of infectious disease using an analysis of how infectious disease spreads [5]. COVID-19 is highly contagious, and since no specific treatment has been developed yet for it [6], using modeling tools to identify the virus-infected areas can help prevent its spread. However, there have been few risk maps of the virus-infected urban areas to prevent travel to risky places.

Many factors influence the transmission of a virus [7]. Since the spread of epidemic urban diseases is strongly affected by urban activities [8], identifying the relationships between the different urban land uses and the transmission of COVID-19 is an effective tool to prevent its spread across the city. Geographic information system (GIS) has long been considered by health experts to be an important tool in the prevention and control of infectious diseases. With the invention of computerized GIS, its use with analyzing, visualizing, and the discovery of disease-spreading patterns has increased significantly [1]. Therefore, using GIS, the distribution of urban land uses and the relationship between the distribution of these land uses and high-risk areas of the disease can be determined.

With the ability to quickly analyze big data and understanding the epidemic rules, GIS supports preventive decisions and actions [9]. So far, GIS-based studies have been conducted to spatial analyze COVID-19. In the continental United States, Mollalo et al. [10] examined GIS-based spatial modeling of the COVID-19 incident rate using a geographically weighted regression (GWR) model. Kanga et al. [11] assessed the risk of COVID-19 contagion using remote sensing and GIS analysis. Urban et al. [12] used GIS-based spatial modeling of COVID-19 deaths in Sao Paulo, Brazil, using the GWR model. Through Bayesian probabilistic modeling and the GIS-based Voronoi method, Bherwani et al. [13] investigated the understanding of COVID-19 transmission. In this study, the relationship between the population in each polygon and the COVID-19 prevalence rate was investigated. Bag et al. [14] examined the understanding of the spatio-temporal pattern of COVID-19 prevalence in India using GIS and the Moran spatial autocorrelation index. Silalahi et al. [15] examined GIS-based approaches to referral hospital access using network analysis and the spatial distribution model of COVID-19 disease spread in Jakarta, Indonesia. Rahman et al. [16] investigated GIS-based spatial modeling to identify the factors influencing the incidence of COVID-19 in Bangladesh using a spatial regression algorithm. Razavi-Termeh et al. [17] examined Iran’s vulnerability to COVID-19 using four criteria: population density, percentage of elderly people, temperature, and humidity.

Research on the spatial analysis of COVID-19 disease has so far made little mention of urban land uses and their role in the spread of the COVID-19. The density of urban land uses and incompatibility in them can lead to population density and greater prevalence of COVID-19. Therefore, one of the objectives of this study was to investigate the correlation and relationship between land uses with COVID-19 and their use in preparing a risk map. Owing to the nature of the data, machine learning algorithms were used to prepare the COVID-19 risk map. Relationships in the real world are nonlinear, which has led to the widespread use of machine learning algorithms in various sciences that examine the nonlinear and complex relationships between observations and prediction variables [18]. Machine learning algorithms enable the analysis of big data. In addition, they usually allow easier, more accurate outcomes to predict. A core element of spatial analysis in GIS is machine learning [19]. Since the purpose of this study is to predict COVID-19 risk with respect to urban land uses, the regression algorithm of machine learning algorithms was used. To predict the COVID-19 risk among machine learning algorithms, three categories of machine learning regression algorithms, including neural networks (adaptive neuro-fuzzy inference system (ANFIS) algorithm), decision trees (random forest (RF) algorithm), and generalized linear algorithms (logistic regression (LR) algorithm), were used. These three algorithms have shown their ability in GIS-based environmental modeling [20,21,22,23,24]. Although useful studies have been performed on the spatial analysis of COVID-19 using GIS, few studies have prepared a COVID-19 risk map in urban environments using machine learning algorithms. To the best of the authors’ knowledge, the impact of socio-economic land uses on the modeling of COVID-19 has not been used so far, and this study offers an approach to reduce population density in socio-economic land uses. 

## 2. Materials and Methods

### 2.1. Methodology

**Figure 1:**
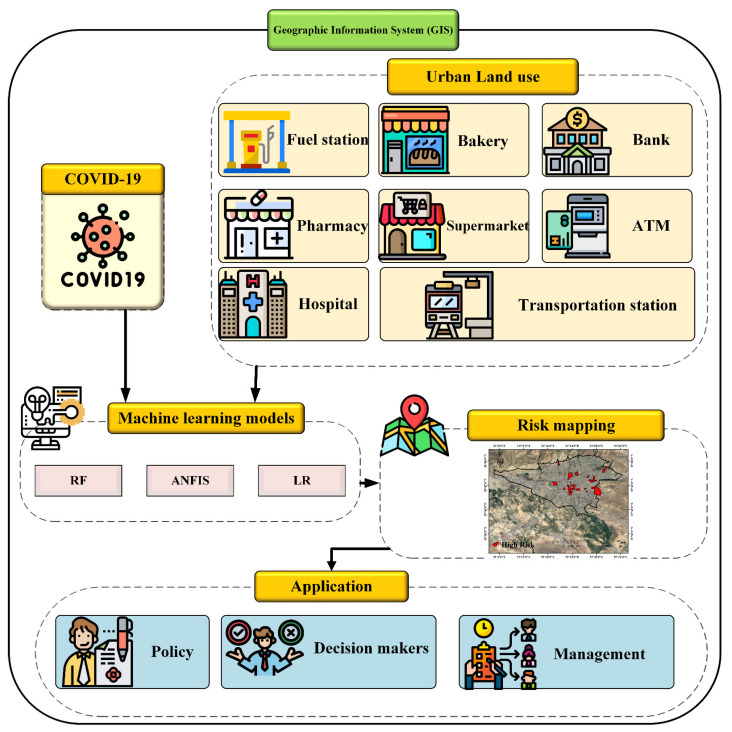
Research framework.

In Figure 1, the research framework is shown and contains the following steps:

Step1: Creating a spatial database including the location of patients with COVID-19 and urban land use.

Step 2: Modeling of COVID-19 disease risk areas using machine learning algorithms (RF, ANFIS, and LR algorithms) in the MATLAB R2017b software (Mathworks, Natick Massachusetts, United States) and determining the importance of variables using the OneR technique.

Step 3: Mapping the high-risk areas of COVID-19 disease and evaluating the results.

Step 4: Provision of solutions to determine management policies and decision makers in the control of COVID-19 disease.

### 2.2. Study Area

**Figure 2:**
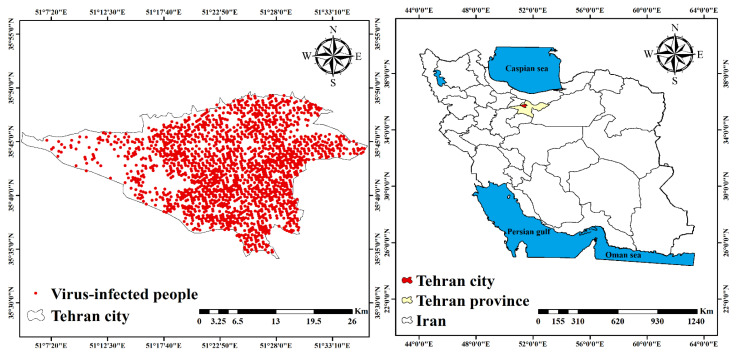
Distribution of COVID-19 in Tehran.

Tehran is the capital of Iran, which has an approximate area of 730 km^2^. The Tehran city is located between the latitude of 51°6′ to 51°38′ N and the longitude of 35°34′ to 35°51′ E. The population of Tehran is 8,693,706, and Tehran is the largest and most populous city in Iran, according to the most current official statistics from the Statistics Center of Iran in 2016. Additionally, more than 10% of Iran’s population lives in this city. On 21 February 2020, the first patient with the virus was identified in Tehran, and the number of patients in this city reached 2282 by 21 March 2020 according to the Ministry of Health and Medical Education of Iran, which showed that Tehran is the most infected city in Iran with COVID-19. In order to model the COVID-19 risk, the patients’ residential addresses were used, and this information was converted into point data. Figure 2 shows the distribution map of COVID-19 patients in Tehran by 21 March 2020. In order to perform better with the modeling, the same number of COVID-19 non-occurrence data (value 0) was randomly generated in addition to the COVID-19 related training data (value 1) (2282 points).

### 2.3. The Effective Criteria 

**Figure 3:**
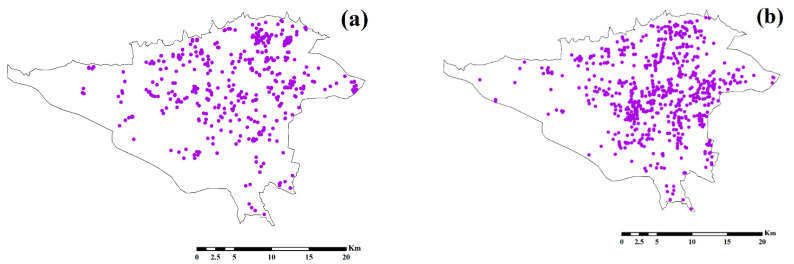

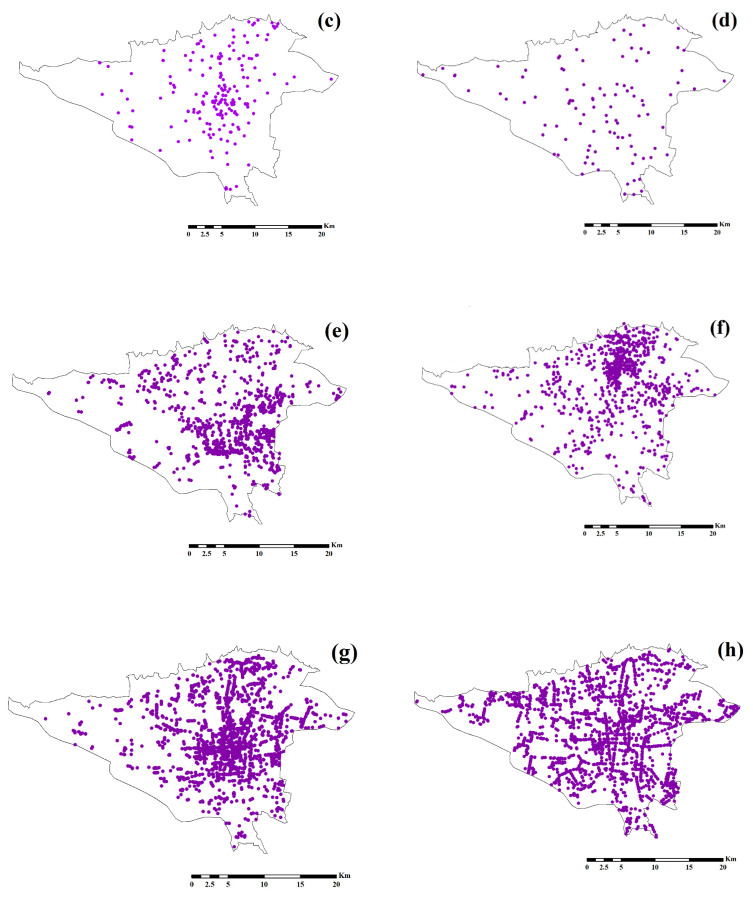
Location map of the public urban land uses in Tehran: (**a**) supermarket, (**b**) pharmacy, (**c**) hospital, (**d**) fuel station, (**e**) bakery, (**f**) ATM, (**g**) bank, and (**h**) public transportation station.

Social activity has a direct effect on the spread of COVID-19, and the transmission of this virus is still ongoing until all its activity is reduced [25]. Quarantine on a personal and social level is effective in controlling COVID-19 [26]. Urban quarantine and restrictions on urban activities have been shown to be successful in decreasing the spread of COVID-19 [27]. However, even during the quarantine period, all urban activities did not stop completely, and some activities continued. In this research, eight public urban land uses were selected that continued to work during the quarantine as effective sites for the COVID-19 outbreak, which included automated teller machines (ATMs), bakeries, banks, fuel stations, hospitals, pharmacies, public transportation stations, and supermarkets. Open Street Map (OSM) (https://www.openstreetmap.org/, accessed on 2 February 2020) data was used to prepare the spatial data set of the effective criteria in Tehran. The numbers of land uses in the study area are presented in Table 1. Figure 3 shows the distribution map of each criterion. Land use density was obtained using kernel density analysis in ArcGIS 10.3 (ESRI, Redlands, CA, USA) to analyze the impact of each land use on COVID-19 modeling. Using Equation (1), the kernel density was calculated [28]:

(1) $$Density=\frac{1}{{\left(radius\right)}^{2}}\overset{n}{\underset{i=1}{\sum }}\left[\frac{3}{\pi }pop_{i}{\left(1-{\left(\frac{dist_{i}}{radius}\right)}^{2}\right)}^{2}\right]$$

where *i* = 1, …, *n* are the input points, *pop_i_* is the population field value of point *i*, and *dist_i_* is the distance between point *i* and the (*x*, *y*) location. During the day, ATMs are touched by many people, which makes these devices much polluted [29]. Iranians consume an average of five times more bread than Europeans per day [30]. Therefore, bakeries are one of the busiest places in Iran. Since the main method of COVID-19 transmission is human-to-human transmission [31] and the prevalence of this virus in crowded places is significantly high [32], bakeries can be considered as the centers of COVID-19 outbreak in Iran. Banks, which include both a place to exchange and hold cash and a busy public place, are at high risk for the COVID-19 outbreak [29]. With regard to hospitals, human-to-human hospital-associated transmission of COVID-19 is the cause of the infection of a significant percentage of the patients. After the virus spread, they have become known as a potential source of COVID-19 transmission through contact with contaminated surfaces [33]. The direct connection of infected people with pharmacies has made them one of the most dangerous places to the extent that various protocols have been proposed by the relevant organizations in order to increase the safety of the pharmacy staff [34]. Public transport stations: there is a significant relationship between the use of public transport and the spread of COVID-19, so much so that the ban of its use was considered as a quick policy to prevent the further spread of the virus [35]. Finally, supermarkets are among the busiest places during the virus outbreak, and several clusters of supermarket workers can be seen among the infected for this reason [36].

In the first step, the density map of each socio-economic criterion was prepared and in order to eliminate the uncertainty. All the density maps were fuzzy using the linear membership function. The fuzzy maps for the socio-economic criteria and the training data related to the COVID-19 were used for the modeling. For this purpose, all points 1 and 0 were extracted from the values of fuzzy maps and considered as input. From the data, 70% were used as training data and 30% as test data, randomly.

### 2.4. Methods

#### 2.4.1. The RF Algorithm

The RF is one of the algorithms for supervised machine learning that utilizes a group of decision trees to predict a sample [37]. A large number of decision trees are created in this algorithm, and the algorithm selects the decisions with the most votes. The bagging process in this algorithm is considered to generalize the results. To create each tree in this algorithm, a different set of existing patterns is determined by considering the replacement of each selected pattern. Independently of the previous random vectors, a random vector (socio-economic land uses) is generated in the RF algorithm and distributed to all trees. Each tree is initially randomly selected using only some data points, then in each division, only a random selection of possible variables is considered. The RF consists of two trees (two classes) in this research (COVID-19 and non-COVID-19 locations), and each was created using eight random features (socio-economic land uses) [38]. To increase the predictive power of this algorithm, the correlation between the trees should be reduced, and the strength of trees should be increased [39].

#### 2.4.2. The LR Algorithm

LR is one of the multivariate mathematical methods of regression. In this mathematical model, the independent variables (socio-economic land uses) are used to predict the probability of the bivariate dependent variables (COVID-19) [40]. LR tries to obtain the best fitting model for the COVID-19 risk map to describe the relationship between the COVID-19 and socio-economic land uses [41]. The LR algorithm can be defined as the following Equation (2):

(2) $$p=\frac{1}{1+e^{-z}}$$

p∈[0,1] and z∈(−∞,+∞)

In Equation (2), *p* is the probability of an event occurring. The *z* parameter, which is calculated according to Equation (3), is a linear set of constant values [41].

(3) $$z=\beta_{0}+\beta_{1}x_{1}+\beta_{2}x_{2}+\beta_{3}x_{3}+\ldots +\beta_{n}x_{n}$$

where *β*_0_ has a constant value, *β*_1_ … *β_n_* are regression coefficients, and *X*_1_ … *X_n_* are independent variables.

#### 2.4.3. The ANFIS Algorithm

To build the ANFIS algorithm, artificial neural networks (ANN) and fuzzy logic (FL) are combined. The purpose of this combination is that ANFIS uses the benefits of both ANN and FL in one framework [42]. Even though fuzzy algorithms can describe complex processes using IF-THEN rules, they are not capable of automatic training. Additionally, it becomes very difficult to select the appropriate membership functions and the if-then rules for the fuzzy model as the number of input variables of a problem increases. In contrast, even though the ANN algorithms can automate training, they cannot describe the system and how to achieve the results [43].

**Figure 4:**
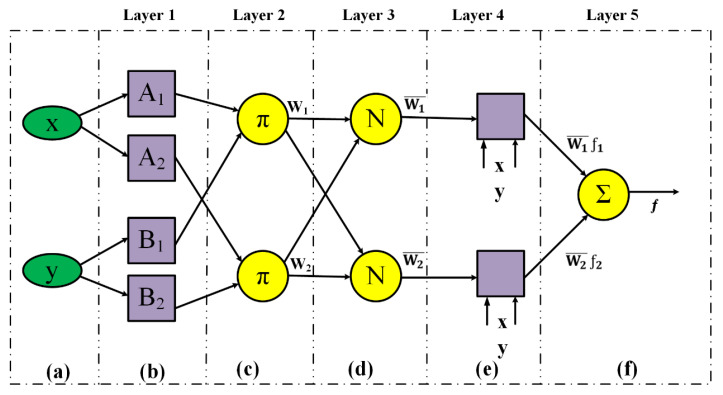
ANFIS layers: (**a**) input factors, (**b**) fuzzification, (**c**) rules, (**d**) normalization, (**e**) defuzzification, and (**f**) aggregation [42].

The ANFIS algorithm consists of 5 layers (Figure 4), which are as follows [44]: 

All the input nodes in the first layer are the adaptive nodes Equations (4) and (5):

(4) $$O_{1,\;\;i}=\mu A_{i}\left(x\right)$$

(5) $$O_{1,\;\;i}=\mu B_{i}\left(y\right)$$

In Equations (4) and (5), *A* and *B* are the linguistic variables, and *μA_i_*(*x*) and *μB_i_*(*y*) are the membership functions of the input nodes *x* and *y*.

Layer 2 has constant nodes as *π*. Every node with the role as a fuzzy AND action is used for the firing strength computation of the rules as the output layer. All the input signals to a node produce the output of each node Equation (6):

(6) $$O_{2,\;i}=W_{i}=\mu A_{i}\left(x\right)\;\mu B_{i}\left(y\right),\;i=1,2$$

where *W_i_* is the output of each node.

Layer 3 consists of a set of fixed nodes with the symbol N. The nodes in this layer are normalized to the firing strength from the second layer, which is known as the normal firing power Equation (7):

(7) $$O_{3,\;i}=W_{l}=\frac{w_{i}}{w1+w2},\;i=1,2$$

Each node in the fourth layer is linked to a node function Equation (8):

(8) $$O_{4,\;i}=\bar{W_{l}}\;f_{i}=\bar{W_{l}}\left(p_{i}x+q_{i}y+r_{i}\right),\;i=1,2$$

where $\bar{W_{l}}$ is the normalized firepower of the third layer, and *p_i_*, *q_i_*, and *r_i_* are the linear parameters.

Layer 5 is the output layer, and it contains a single node with the symbol ∑. This layer is the sum of all the inputs from layer 4 and is equal to the final result of the algorithm Equation (9):

(9) $$O_{5,\;i}=\sum \bar{W_{l}}\;f_{i}=\sum w_{i}\;f_{i}/\sum w_{i},\;i=1,2$$

#### 2.4.4. Feature Selection Using OneR Technique

In this study, the OneR technique was used to investigate the importance of variables in modeling. This method examines the correlation between patients’ geographical location and variables and assigns importance to each variable based on the weight of the correlation obtained. This method is also used to check whether all variables can participate in modeling. The OneR approach is a one-tier decision tree that includes a series of rules in the dataset that all evaluate a particular property. The OneR approach is simple and also offers good rules for data structures to be characterized. To obtain the weight of each effective criterion, the OneR strategy uses the computational error ratio and other rules [45]. 

#### 2.4.5. Pearson Correlation Technique

A measure of the linear dependence between two random variables is the Pearson correlation coefficient. Pearson correlation coefficient between two variables is calculated by dividing their covariance by standard deviations. Pearson correlation between *x* and *y* variables was calculated using Equation (10) [46].

(10) $$r_{xy}=\frac{\sum \left(x_{i}-\bar{x}\right)\sum \left(y_{i}-\bar{y}\right)}{\sqrt{\sum {\left(x_{i}-\bar{x}\right)}^{2}}\sqrt{\sum {\left(y_{i}-\bar{y}\right)}^{2}}}$$

where $\bar{x}$ denotes the mean of *x*, $\bar{y}$ denotes the mean of *y*, and $r_{xy}$ is the Pearson coefficient.

#### 2.4.6. Validation

To test the modeling, the ROC curve was used. There are sensitivity axes (*x*-axis) and a transparency axis (*y*-axis) in the ROC curve. The *x*-axis and the *y*-axis for the ROC curve are calculated using Equations (11) and (12) [47,48].

(11) $$X=1-\left[\frac{\mathrm{TN}}{\mathrm{TN}+\mathrm{FP}}\right]$$

(12) $$Y=\left[\frac{\mathrm{TP}}{\mathrm{TP}+\mathrm{FN}}\right]$$

The area under the ROC curve, known as the AUC, describes the importance of the prediction of a system by defining its capacities to correctly forecast the occurrence of an event and its non-occurrence [42]. The root mean square error (RMSE) and the mean absolute error (MAE) indices were used to calculate the prediction error Equations (12) and (13).

(13) $$\mathrm{RMSE}=\sqrt{\frac{\sum_{i=1}^{n}{\left(Y-Y\prime \right)}^{2}}{n}}$$

(14) $$\mathrm{MAE}=\frac{1}{n}\sum_{i=1}^{n}\left|Y-Y\prime \right|$$

Y is the real value, Y′ is the predicted value, and n is the number of samples [48].

## 3. Results

### 3.1. Feature Selection

**Figure 5:**
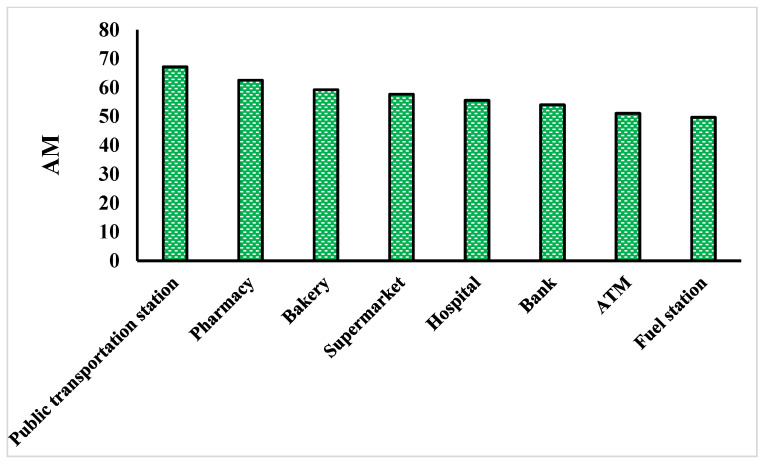
Result of OneR method.

Feature selection results using the OneR method are shown in Figure 5. This finding reveals that in the COVID-19 risk mapping, all eight parameters have significance (average merit (AM) > 0). On the basis of the OneR technique performance, criteria of public transportation station (67.18), pharmacy (62.53), bakery (59.27), supermarket (57.7), hospital (55.58), bank (54.01), ATM (51.08), and fuel station (49.71) are the most important on COVID-19 risk modeling. The results show that all variables can participate in modeling.

### 3.2. Correlation between COVID-19 and Land Use

**Figure 6:**
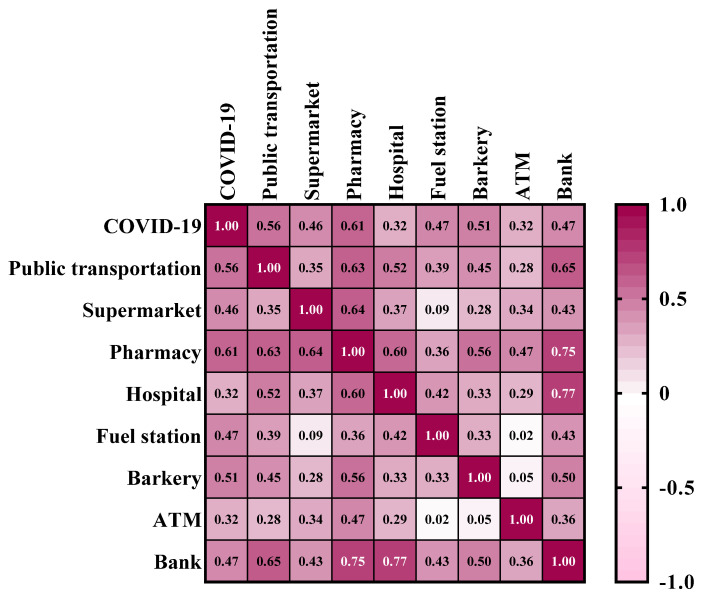
Results of the Pearson correlation.

The results of the Pearson correlation between COVID-19 and land uses are shown in Figure 6. According to the results, COVID-19 have the highest correlation with public transport stations (0.56) and pharmacies (0.61). According to the results, the land uses of the public transport stations (0.65), pharmacies (0.75), hospitals (0.77), and fuel stations (0.43) are most correlated with the land use of the bank. Additionally, land uses of supermarkets (0.64), bakeries (0.56), and ATMs (0.47) are most correlated with the land use of pharmacies. The results showed that the density of bank and pharmacy land uses is most correlated with other land uses in the prevalence of COVID-19.

### 3.3. COVID-19 Modeling Process

**Figure 7:**
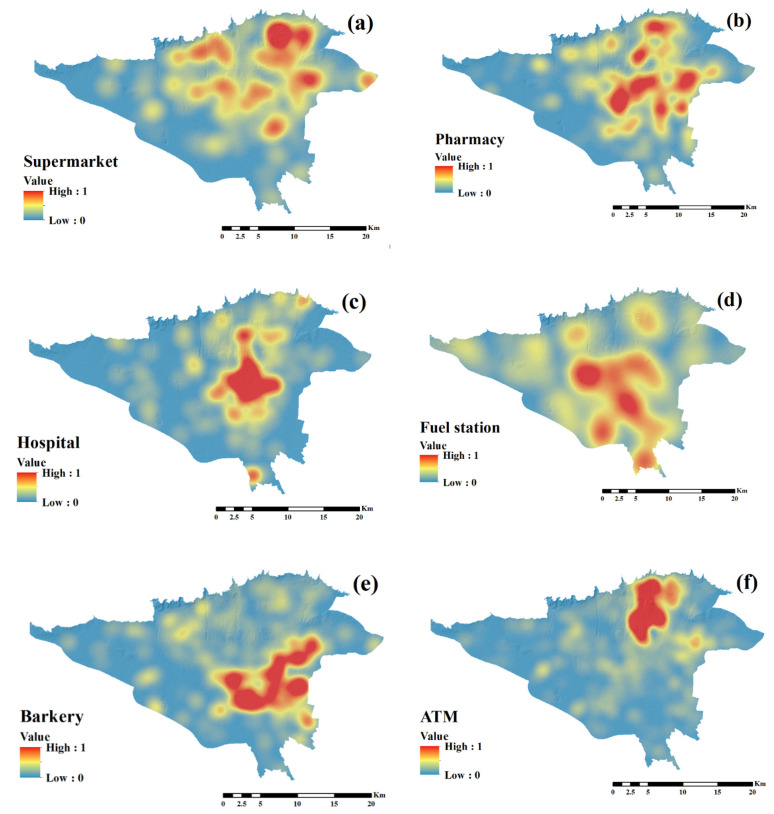

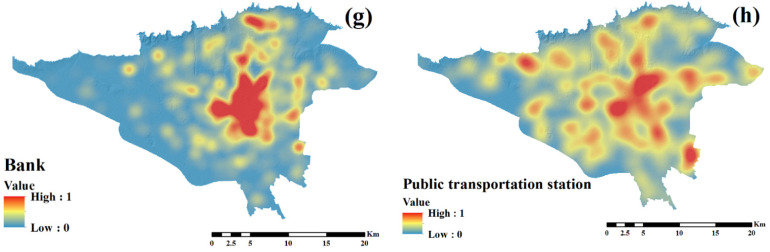
Fuzzy maps of socio-economic land use: (**a**) supermarket, (**b**) pharmacy, (**c**) hospital, (**d**) fuel station, (**e**) bakery, (**f**) ATM, (**g**) bank, and (**h**) public transportation station.

The spatial database used as input to the machine learning algorithms included dependent data (COVID-19 patient’s location (1) and COVID-19 patient’s non-location (0)) and independent data (land use fuzzy maps (Figure 7)). The input matrix of machine learning algorithms consists of nine columns (eight columns equal to land uses and the last column of the target (values 0 and 1)) and 4564 rows (2282 rows of COVID-19 patients and 2282 rows of patients without COVID-19).

**Figure 8:**
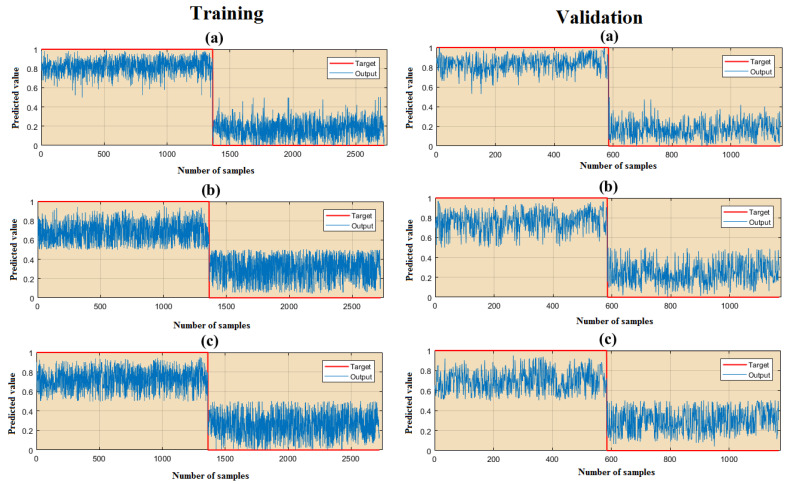
The results of the training and validation data for the (**a**) RF, (**b**) LR, and (**c**) ANFIS.

The results of the ability to predict the three algorithms and the modeling output on the training and the validation data are shown in Figure 8. The results for the RMSE and MAE values of all three algorithms for the training data and the validation data are presented in Table 2. 

According to the results of the training and validation data, the lowest value for the RMSE is related to the RF (0.1963 and 0.549), ANFIS (0.277 and 0.557), and LR (0.365 and 0.571) algorithms. The findings of the MAE index showed that RF (0.176 and 0.511), ANFIS (0.2511 and 0.52), and LR algorithms (0.33 and 0.526) were the lowest values for this index.

**Figure 9:**
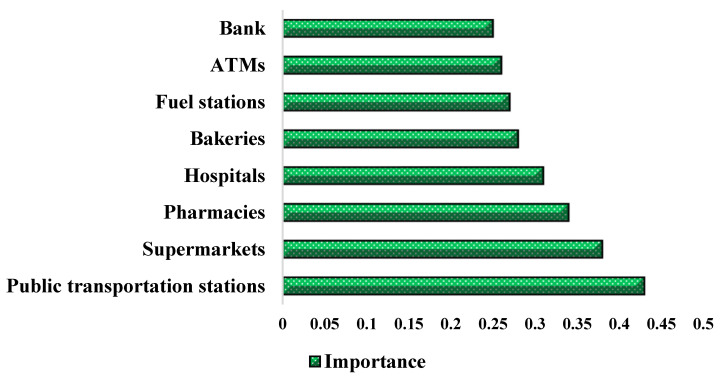
The importance of the socio-economic criteria by the RF algorithm.

In Figure 9, the significance of each of the effective parameters using the RF algorithm is shown. According to the results, the most important are public transport stations (0.43), supermarkets (0.38), pharmacies (0.34), hospitals (0.31), fuel stations (0.28), bakeries (0.27), ATMs (0.26) and banks (0.25). 

The results of the LR algorithm are given in Table 3. According to the results, pharmacies (0.899), public transport stations (0.794), fuel stations (0.747), hospitals (0.515), supermarkets (0.499), bakeries (0.4), banks (0.075), and ATMs (0.057) are positively related to the COVID-19 disease. 

**Figure 10:**
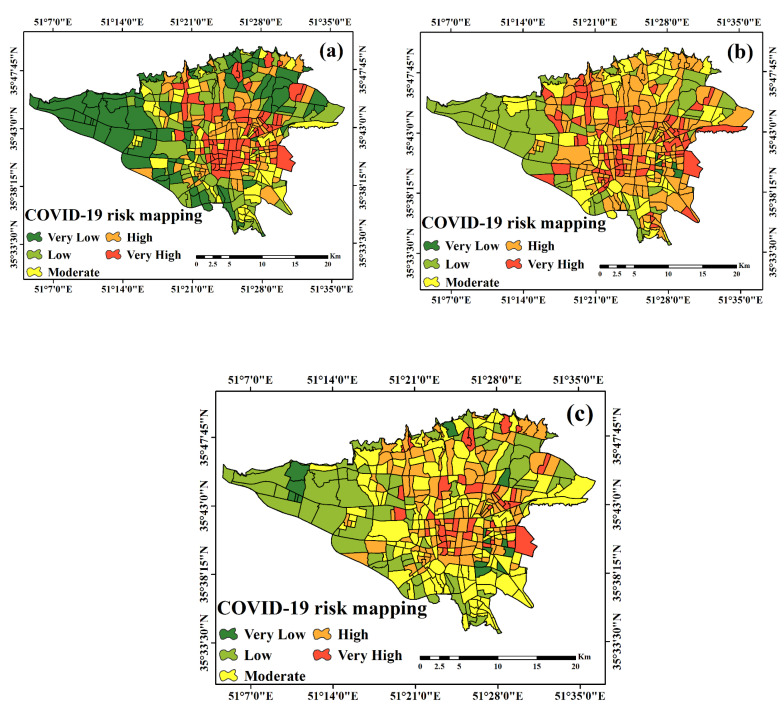
COVID-19 risk mapping by (**a**) RF, (**b**) ANFIS, and (**c**) the LR algorithms.

**Figure 11:**
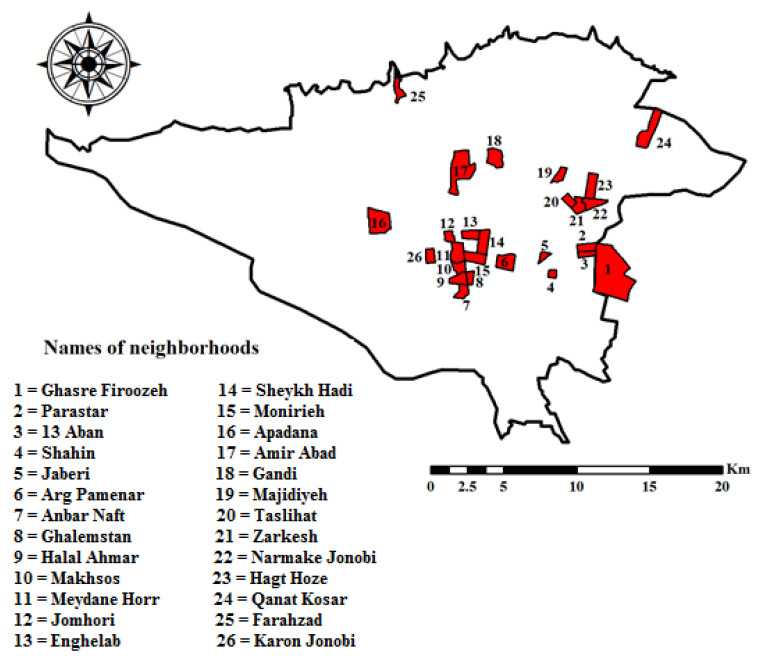
High-risk areas by the three algorithms.

After training the algorithms, the fitted model was generalized to the whole study area, and the COVID-19 disease risk map in Tehran was prepared using the three algorithms in ArcGIS 10.3 software. The classification of the maps was based on the natural break method and was divided into five categories, which included very low, low, medium, high, and very high risk (Figure 10a–c). According to the results of the three algorithms, the highest vulnerability is related to the central areas of Tehran. Vulnerability in the middle areas of Tehran in the RF algorithm is more than the other two algorithms. In the RF and LR algorithms, high-risk areas are less scattered than the ANFIS algorithm. In the LR algorithm, the number of high-risk areas is lower than the other two algorithms. Areas with high risk are shown in Figure 11 using the results of the three algorithms. According to the results, the central and the eastern areas of Tehran have more vulnerabilities than the other areas. The central areas of Tehran have a higher population density, and Tehran is where the most important commercial and economic centers are located. Therefore, due to the social interaction of most people in these areas, it is one of the high-risk areas for the COVID-19 disease. 

### 3.4. Validation of COVID-19 Risk Maps

**Figure 12:**
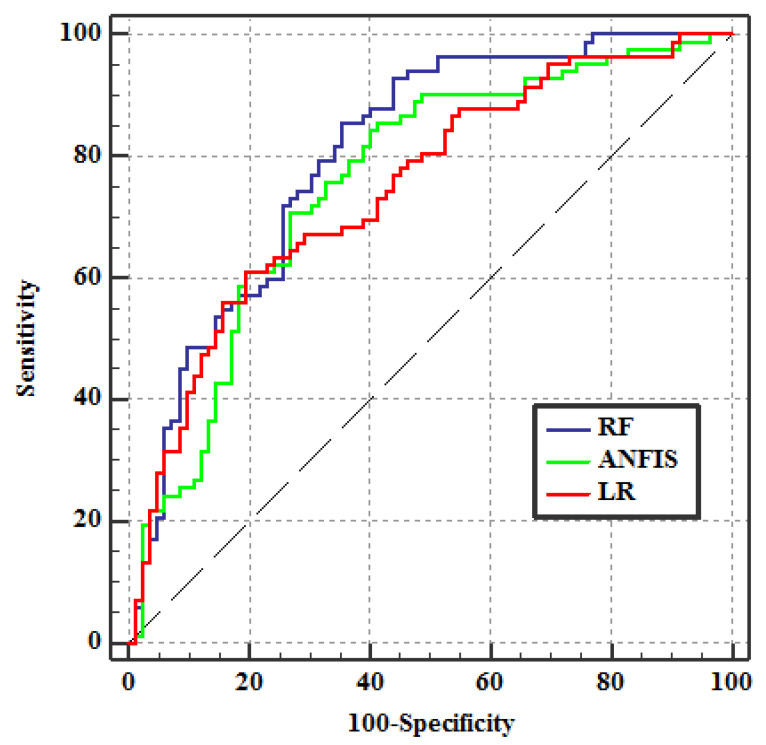
Results of the ROC curve.

To evaluate the final risk maps, 30% of COVID-19 disease data (occurrence (value 1) and non-occurrence (value 0)) were extracted from three risk maps. The validation results from the ROC curve and the AUC are shown in Figure 12 and Table 4. The AUC value of the prediction rate curve is 0.803, 0.758, and 0.747 for the RF, ANFIS, and LR algorithms, respectively. The results showed that the RF algorithm had a higher accuracy than the ANFIS and LR algorithms with COVID-19 risk mapping.

To examine pairwise differences between the algorithms, the Wilcoxon signed-rank test was used. When the *p* value is less than 0.05 and the Z value is more than −1.96 and +1.96, the algorithms’ capacity is predicted to be significantly different [49]. The Z values and *p* values for each pairwise comparison of RF-ANFIS, RF-LR, and LR-ANFIS exceeded the critical thresholds of ± 1.96 and 0.05, indicating significant statistical differences among the models employed in this study (Table 5).

## 4. Discussion

The city of Tehran is one of the main centers of COVID-19 in Iran due to its high population and the location of important economic, social, political, and other centers in this city. In densely populated urban centers, some urban land features that citizens use frequently can be a focal point for the spread of COVID-19. The purpose of this study is to map the risk of the urban areas against COVID-19 according to the socio-economic land uses and three machine learning algorithms, which include RF, ANFIS, and LR. According to the ROC results, the RF algorithm had a higher accuracy with COVID-19 risk mapping than the other two algorithms. The RF algorithm works well in a data set with missing data [21]. On the other hand, with increased training data in the ANFIS algorithm, the performance of the algorithm improves, but it cannot act effectively like the RF algorithm for low volume data [50]. With regard to the disadvantages of the LR algorithm compared to the RF algorithm, the LR algorithm requires processing with more data volume and inflexibility with a high-level database [51]. Therefore, the use of this algorithm can be effective to prepare the risk map of COVID-19 due to the advantages that are mentioned above in relation to the RF algorithm compared to the other two algorithms. 

According to the results of the RF and the LR algorithms, COVID-19 had the greatest impact on public transport stations and pharmacies in Tehran. Public transport stations are known as one of the outbreak’s major centers for the disease due to the high passenger traffic and overcrowding. This effect is much more noticeable with public transportation because the use of public transportation, such as subways and buses, increases communication and increases the risk of COVID-19. In Tehran, 15 million trips are made daily, nearly 7 million passengers use metro stations daily, and 3 million passengers use buses daily. According to the results, it seems that the high density of the passengers in these stations, the physical contact of the passengers, and the non-compliance with social distancing are the main reasons for the outbreak of COVID-19 in Tehran. Another important center for the spread of COVID-19 in Tehran are pharmacies. Some of the reasons for this are the proximity of these centers to hospitals and the fact that people go to pharmacies to buy masks and gloves, which increases the population density. According to the results, another center that has an impact on the outbreak of COVID-19 are supermarkets, which can affect the outbreak of COVID-19 due to the population of 8 million people in Tehran, and the demand of people to buy food in these centers. The city of Tehran has almost 4 million cars and more than 3 million motorcycles, and the daily need of these devices is fuel, which increases the population density at fuel stations and can be instrumental in the spread of COVID-19.

If land uses are distributed in cities in a way that causes decentralization, the vulnerability to the disease can be expected to be greatly reduced. Two important concepts in urban land use in relation to the disease include compatibility and proximity. In terms of compatibility, the two land uses of banks and pharmacies were most correlated with other land uses. It seems that these two land uses in the study area did not have good distribution, and the interference of these two land uses was not compatible with other land uses and caused the concentration of population in these land uses. Therefore, relocation of these two land uses in high-risk areas of the disease can reduce the population and reduce the spread of the virus. In such circumstances, the pattern of the normal distribution of resources should be abandoned, and urban resources should be distributed in proportion to the level of vulnerability of neighborhoods. 

Therefore, risk maps prepared using GIS can significantly help officials and individuals make special arrangements in regard to high-risk areas, and they can reduce the outbreak of COVID-19 in these areas while maintaining social distancing. 

One of the disadvantages of this study was the lack of access to accurate polygon data of land use. Other urban land uses, such as commercial, parks, industrial, and administrative centers, can also be utilized to investigate more comprehensively and obtain higher modeling accuracy. Owing to the fact that population density varies at different times of the day, it is suggested that spatio-temporal modeling be used in future research. Additionally, due to the large volume of data, it is suggested to use deep learning algorithms for modeling in future research.

## 5. Conclusions

This study examined an approach that combined machine learning, GIS, and urban land use to prepare a COVID-19 risk map. The results showed that the machine learning algorithms had good accuracy in preparing the COVID-19 risk map, while the RF algorithm had a higher accuracy. The results showed that the urban land use of public transportation stations, pharmacies, and supermarkets had a greater effect on the prevalence of COVID-19 in Tehran. It seems that due to the high use of these land uses and the increase in population density in them, the prevalence of COVID-19 in these areas is higher. COVID-19 risk maps in Tehran showed that the central and eastern regions are more vulnerable due to population density and land use density in these areas. According to the results, the distribution of the two land uses of pharmacies and banks causes incompatibility with other land uses, increases the concentration of the population in these land uses, and increases the spread of the virus. The map of the high-risk areas can help people and managers to manage and reduce the population density in order to reduce the outbreak of COVID-19 in these areas. High-risk area maps can help managers assess land use distribution in critical situations. 

## Figures and Tables

**Table 1:** Number of land uses.

Land Use	Number of Land Uses	Format
ATM	1084	Point
Bank	2378	Point
Bakery	900	Point
Fuel station	102	Point
Hospital	196	Point
Pharmacy	661	Point
Supermarket	443	Point
Public transportation station	2113	Point

**Table 2:** The results of the RMSE and the MAE indices.

	RF		ANFIS		LR	
	Train	Test	Train	Test	Train	Test
RMSE	0.1963	0.549	0.277	0.557	0.365	0.571
MAE	0.176	0.511	0.2511	0.520	0.33	0.526

**Table 3:** The results of the LR algorithm.

Variable	Coefficient	Std. Error
Public transportation stations	0.794	0.434
Banks	0.075	0.42
Pharmacies	0.899	0.276
Fuel stations	0.747	0.214
Bakeries	0.4	0.397
Hospitals	0.515	0.413
ATMs	0.057	0.316
Supermarkets	0.499	0.352
Constant	0.586	-

**Table 4:** Accuracy results of the three algorithms.

Algorithms	AUC	SE	95% CI
RF	0.803	0.0343	0.734–0.861
ANFIS	0.758	0.0381	0.685–0.821
LR	0.747	0.0381	0.673–0.812

**Table 5:** Wilcoxon signed-rank test of the three algorithms.

Pair-Wise Algorithm	Z Value	*p*-Value	Significant
RF-ANFIS	6.3	<0.0001	Yes
RF-LR	5.8	<0.0001	Yes
LR-ANFIS	3.7	<0.0001	Yes

## Data Availability

Data during the current study are not publicly available due to integrity and legal reasons but are available from the corresponding author on reasonable request.
